# Effects of different milk powders on the growth and intestinal flora in weaned rats: Comparison of special formula milk powder with ordinary milk powder

**DOI:** 10.1002/fsn3.4387

**Published:** 2024-11-12

**Authors:** Ruiqi Mu, Yu Fu, Jufang Li, Qinggang Xie, Weiwei Ma

**Affiliations:** ^1^ School of Public Health, Beijing Key Laboratory of Environmental Toxicology Capital Medical University Beijing China; ^2^ Feihe Research Institute Heilongjiang Feihe Dairy Co., Ltd. Beijing China

**Keywords:** growth and development, gut health, infant, intestinal flora, milk powder, short‐chain fatty acid

## Abstract

The objective of this investigation was to examine the effects of distinct dosages of infant formula and diverse formula constituents on the growth and development of weaned rats. Fifty specific pathogen‐free (SPF) male Sprague–Dawley (SD) rats aged 3 weeks were divided into the basic diet group, 20% ordinary milk powder group, 20% special formula milk powder group, 30% ordinary milk powder group, and 30% special formula milk powder group randomly. After 28 days of feeding, compared with the basic diet group, the body mass and brain/body weight of rats in the 30% ordinary and special formula milk powder groups were decreased. At the Genus level, *Bacteroides* in the group supplemented with 20% special formula milk powder was significantly lower than that in the basic diet group, and *Parabacteroide*s was significantly lower than that in the 20% ordinary milk powder group. *Lactobacillus* was significantly higher than those in the basic diet group and the 20% ordinary milk powder group, and *Blautia* was significantly higher than those in the basic diet group and the 20% and 30% ordinary milk powder groups, and *UBA1819* was significantly higher than those in the other groups. The abundance of *Parasutterella* in the basic diet group was significantly higher than those in the groups supplemented with 20% ordinary milk powder, 20% special formula milk powder, and 30% ordinary milk powder. This study found that different doses and different formula components of infant milk powder could affect body mass and intestinal flora in Sprague–Dawley (SD) rats, and the addition of low‐dose (20%) special formula infant milk powder can increase the beneficial bacteria in the intestinal flora of rats and may reduce the pathogenic bacteria.

## Graphical abstract

Different doses and different formula components of infant milk powder could affect body mass and intestinal flora in SD rats. The addition of low‐dose (20%) special formula infant milk powder can increase the beneficial bacteria in the intestinal flora of rats and may reduce the pathogenic bacteria.

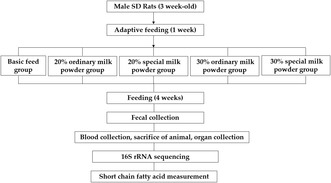

## 1 INTRODUCTION

It is widely known that mother's milk is the best source of nutrition for babies (Lessen & Kavanagh, 2015). Several studies have shown that breast milk contains many components that support the growth, health, and development of infants. In particular, components such as maternal antibodies are passed on to the infant through breast milk, shaping the newborn's immunity and influencing its intestinal flora (Atyeo & Alter, 2021; Melnik et al., 2021). However, the decision to breastfeed is very subjective, generally subject to a variety of factors, and sometimes breastfeeding is unable, inappropriate, or insufficient and has to be interrupted. Worldwide, only 38% infants are breastfed alone, the prevalence is only about 30% in most developing world countries (Melese Ayele, 2021). Therefore, infant formula is a great alternative to breastfeeding. Although it is highly unlikely to produce products that are identical to breast milk, researchers are trying their best to minimize the nutritional gap between the product and breast milk in order to promote normal growth and development of infants (Liu, Jiang, et al., 2023). Soy milk or milk is typically utilized as the foundational element of formula, with additional components incorporated to closely mimic the makeup of human breast milk and garner nutritional advantages (Liu, Lei, et al., 2023). These supplements may consist of iron, a blend of nucleotides and fats, and essential fatty acids like arachidonic acid (ARA) and docosahexaenoic acid (DHA). Diana Cowland, an analyst, predicts that the fast growth of baby formula will be fueled by Asia, particularly China, and is projected to persist with a compound annual growth rate of 11% (Martin et al., 2016).

It is now generally accepted that the first 1000 days of life, from conception to 2 years postpartum, are a uniquely important period in infant development (Christifano & Bennett, 2023; Martorell, 2017). This stage is the period when the infant microbiome rapidly matures. The infant gut microbiota contributes to the protection of infants against harmful pathogens, and gut probiotics play a crucial role in the formation of the immune system, especially in protection against inflammatory diseases (Ames et al., 2023; Aziz, Khan, et al., 2023; Yao et al., 2021). The nutritional status of infants influences their early developmental processes, and a proper diet promotes the maturation of the infant's immune system and directly influences the composition and metabolism of the intestinal microbiota to be consistent with that of a healthy adult. (Flint et al., 2012; Matsuyama et al., 2019). Meta‐analysis shows a significant difference in infant gut microbiota between infants that are breastfed and formula‐fed (Ho et al., 2018). Accordingly, it is greatly essential to explore the nutritional factors in breast milk that are beneficial to gut health and use them to improve formulas.

This experiment intends to compare the biological indicators of feeding special infant formula with different formulations to weaned rats, so as to explore whether the ingredients of different infant formula meet the basic nutritional needs of infants and whether the special formula is more beneficial to growth and development. It provides clues for the follow‐up epidemiological study and provides evidence for improving the development of infant.

## 2 MATERIALS AND METHODS

### 2.1 Animals and feeding

**FIGURE 1:**
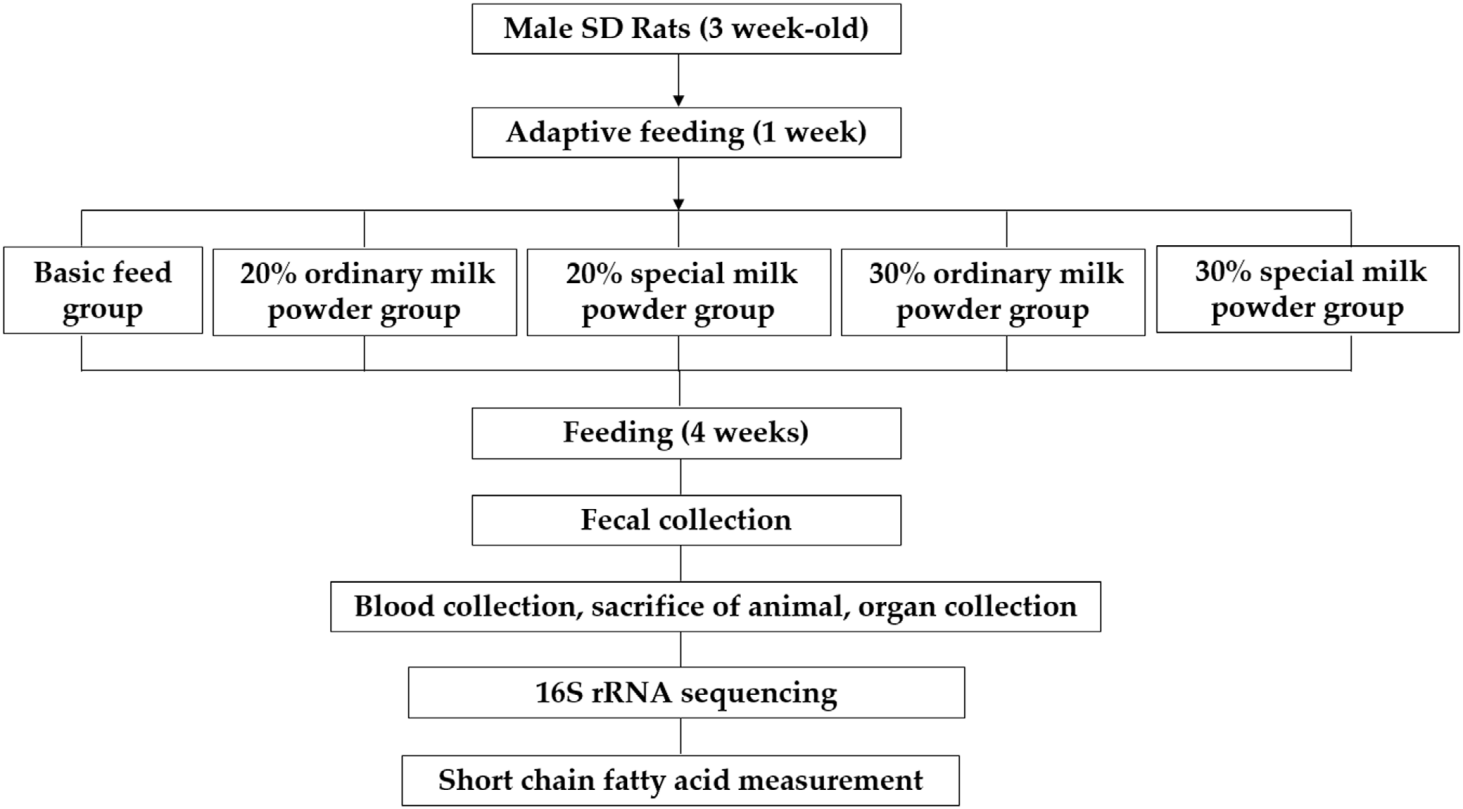
The flowchart of experimental design.

All experimental protocols of use and care of experimental animals were approved and adopted by the Ethics Committee of Capital Medical University (Ethics Review No: AEEI‐2023‐035). Fifty specific‐pathogen free (SPF) male Sprague–Dawley (SD) rats aged 3 weeks after weaning (Beijing, China, SCXK‐(Jing)‐2021‐0011) were fed for 7 days, and randomly divided into five groups according to body weight, including basic diet group, 20% ordinary milk powder group, 20% special formula milk powder group, 30% ordinary milk powder group, and 30% special formula milk powder group, and fed for 4 weeks. Special formula refers to milk powder with appropriate amount of docosahexaenoic Acid (DHA), eicosatetraenoic acid (ARA), galactooligosaccharide (GOS), 1,3‐dioleic acid‐2‐palmitate glycerol triglyceride, lutein, nucleotide, lactoferrin, and casein phosphopeptides, while ordinary milk powder in this experiment did not have the above‐mentioned special ingredients added. The feed is provided by Beijing Keao Xinli (Tianjin) Feed Co., LTD. Beijing, china Ordinary milk powder and special formula milk powder (added with DHA, ARA, galactooligosaccharide, 1,3‐dioleic‐2‐palmitate triglyceride, lutein, nucleotide, lactoferrin, and casein phosphopeptides [mg]) are replaced with corn starch components at two different doses of 20% and 30%. The energy ratio and energy density of the three major macronutrients are as given in Table 1. The specific feed formula is shown in Table 2. The flowchart of experimental design is shown as Figure 1.

**TABLE 1:** Basic diet and experimental diet.

	Basic diet	Ordinary milk powder diet (20%)	Special milk powder diet (20%)	Ordinary milk powder diet (30%)	Special milk powder diet (30%)
Protein	17.3	17.3	17.3	17.3	17.3
Fat	18.8	20.4	21.1	19.5	19.8
Carbohydrate	63.9	62.3	61.6	63.2	62.9
kcal/g	3.99	3.92	3.93	3.89	3.90

**TABLE 2:** Ordinary milk powder and special milk powder.

	Special milk powder	Ordinary milk powder
kcal/100 g	464.4	464.8
Protein (g)	15	17.5
Fat (g)	20	19.5
Linoleic acid (g)	1.9	1.75
α‐Linolenic acid (mg)	260	–
Carbohydrate (g)	54.4	56.4
*Vitamin*		
Vitamin A (μg RE)	500	448
Vitamin D (μg)	8	6.23
Vitamin E (mg a‐TE)	4.9	3.7
Vitamin K1 (μg)	45	19
Vitamin B1 (μg)	650	272
Vitamin B2 (μg)	723	272
Vitamin B6 (μg)	600	272
Vitamin B12 (μg)	1.3	0.97
Nicotinic acid (μg)	5000	2685
Folic acid (μg)	50	19
Pantothenic acid (μg)	4300	1712
Vitamin C (mg)	49	44.8
Biotin (μg)	15	9.7
*Minerals*		
Sodium (mg)	110	136
Potassium (mg)	420	448
Copper (mg)	315	175
Magnesium (mg)	42	35
Iron (mg)	6.8	6.23
Zinc (mg)	4	1.9
Calcium (mg)	490	389
Phosphorus (mg)	330	202.4
Iodine (mg)	40	35
Chlorine (mg)	310	292
*Other ingredient*		
Selenium (μg)	12	–
Choline (mg)	120	42.8
Manganese (μg)	37	–
Inositol (mg)	40	25.3
Taurine (mg)	38	19
l‐Carnitine (mg)	11	7.8
Docosahexaenoic acid (DHA) (mg)	50	–
Eicosatetraenoic acid (ARA)	85	–
Galactooligosaccharide (mg)	3	–
1,3‐Dioleic‐2‐palmitate triglyceride (g)	4	–
Lutein (g)	210	–
Nucleotide (μg)	30	–
Lactoferrin (mg)	45	–
Casein phosphopeptides (mg)	40	–

### 2.2 Biological sample collection

After the 29th day of feeding, five rats were selected from five groups randomly and fresh feces were collected by stress defecation method. The collected feces were put into several sterile Eppendorf (EP) tubes and stored at −80°C. On the 35th day of group feeding, the rats were anesthetized by intraperitoneal injection of tribromoethanol, and blood was collected from the heart. After euthanasia, the brain, heart, liver, spleen, kidney, testis, colon, ileum, perirenal fat, peritestis fat, and omental fat were rapidly collected from rats. All organs and fat were rinsed with saline repeatedly, dried with filter paper, weighed, and stored at −80°C for later use (*n* = 10).

### 2.3 Serum extraction

Blood was collected in a test tube free of pyrogen and endotoxin and naturally coagulated at room temperature for 20 minutes. The serum was separated by centrifuge at 3000 *g* for 20 minutes.

### 2.4 Paraffin sides and (Hematoxylin and eosin) HE staining

Paraffin sectioning was first performed. The fresh tissue was fixed for about 24 h, trimed, and put in the dehydration box, which in turn was put into the dehydrator in order to dehydrate with gradient alcohol. The embedding machine was used to embed the wax‐soaked tissue, then the tissue was cooled at −20° freezing table, and after the wax was solidified, the wax block was removed from the embedded frame and repaired. The trimmed wax block was cooled at −20° freezing table, and sections were then made on a paraffin sectioning machine at a thickness of 4 μm. Then, paraffin sections were dewaxed by xylene I, xylene II, 100% ethanol I, 100% ethanol II, and 75% ethanol for 20 min, 20 min, 5 min, 5 min, and 5 min, and then rinsed with tap water. Sections were stained with hematoxylin solution for 3–5 min and rinsed with tap water. Then the sections were rinsed with tap water after treating with hematoxylin differentiation solution. Then, the sections were treated with hematoxylin Scott's Tap Water Bluing and rinsed with tap water. After sections were treated with 85% ethanol for 5 min and 95% ethanol for 5 min, the sections were stained with eosin dye for 5 min. Finally, the tissue sections were dehydrated and sealed, microscopically examined, and the images were captured and analyzed, and Image‐Pro Plus 6.0 software was applied to select five intact villi per section at a 100× scale, and measure the length of the villi (micrometers (μm)) and the depth of the crypt fossa (μm), respectively.

### 2.5 16S ribosomal (rRNA) sequencing

Genomic DNA was extracted from SD rat feces. The polymerase chain reaction (PCR) products were quantitatively detected by QuantiFluor^TM^‐ST blue fluorescence quantification system and mixed according to the sequencing quantity requirements of each sample. The construction of MiSeq library; MiSeq sequencing results; and DNA fragments were used as templates and primers were fixed as base sequences in the chip, and PCR was used to synthesize the target DNA fragments to be detected in the chip. Generate DNA clusters. A laser scan was performed on the surface of the reaction plate to read the nucleotide type polymerized by each template sequence in the first reaction. The sequence of template DNA fragments was obtained by statistical analysis of the collected fluorescence signals.

### 2.6 Short‐chain fatty acid measurement

Twenty mg of feces sample was accurately weighed into a grinding tube and added 800 μL of water containing 0.5% phosphoric acid (H_3_PO_4_). The sample was frozen and ground twice at 50 Hz (Hertz) for 3 min, then followed by ultrasonic treatment for 10 min and centrifugation at 4°C and 13,000 *g* for 15 min. Two hundred μL of supernatant aqueous solution was taken into a centrifuge tube, then 0.2 mL of *N*‐butanol solvent containing internal standard 2‐ethylbutyric acid (C_6_H_12_O_2_) (10 μg/mL) was added for extraction. Finally, the tube was vortexed for 10 s, subjected to ultrasound treatment at low temperature for 10 min, followed by centrifugation at 4°C and 13,000 *g* for 5 min, and the supernatant was carefully transferred to sample vials for analysis.

Gas chromatography–mass spectrometry (GC–MS) analysis was conducted utilizing an Agilent 8890B gas chromatograph coupled with an Agilent 5977B/7000D mass selective detector. The system was equipped with an inert electron impact (EI) ionization source (Agilent, USA) operating at an ionization voltage of 70 eV. Analyte compounds were separated with a HP‐FFAP capillary column, with helium as a carrier gas at a constant flow rate (1 mL/ min). The GC column temperature was programmed to hold at 80°C and rise to 120°C (40°C/min)–200°C (10°C/min), and finally hold at temperature of 230°C for 3 min. The injection volume of samples was 1 μL and introduced in splitting mode (10:1) with the inlet temperature of 180°C. The ion source temperature was 230°C and the quadrupole temperature was 150°C. The scanning mode was selected ion monitoring (SIM). Compounds were identified and quantified by software of Masshunter (v10.0.707.0, Agilent, USA).

### 2.7 Statistical analysis

The SPSS 21.0 software for statistical analysis of data was used to obtain relevant information. In order to analyze the experimental data, descriptive statistical method was adopted, mean and standard deviation (SD was used to represent the quantitative indicators). For a comparison of the five groups of measurement data, one‐way analysis of variance (ANOVA) was used for the data conforming to normality and homogeneity of variance, while the non‐parametric test (Kruskal–Wallis test) was used for the data conforming to non‐normality or heterogeneity of variance, where *p* < .05 indicated that the significance level of the difference was statistically significant. GraphPad Prism 5.0 was used for mapping.

## 3 RESULTS

### 3.1 Body weight of SD rats

**FIGURE 2:**
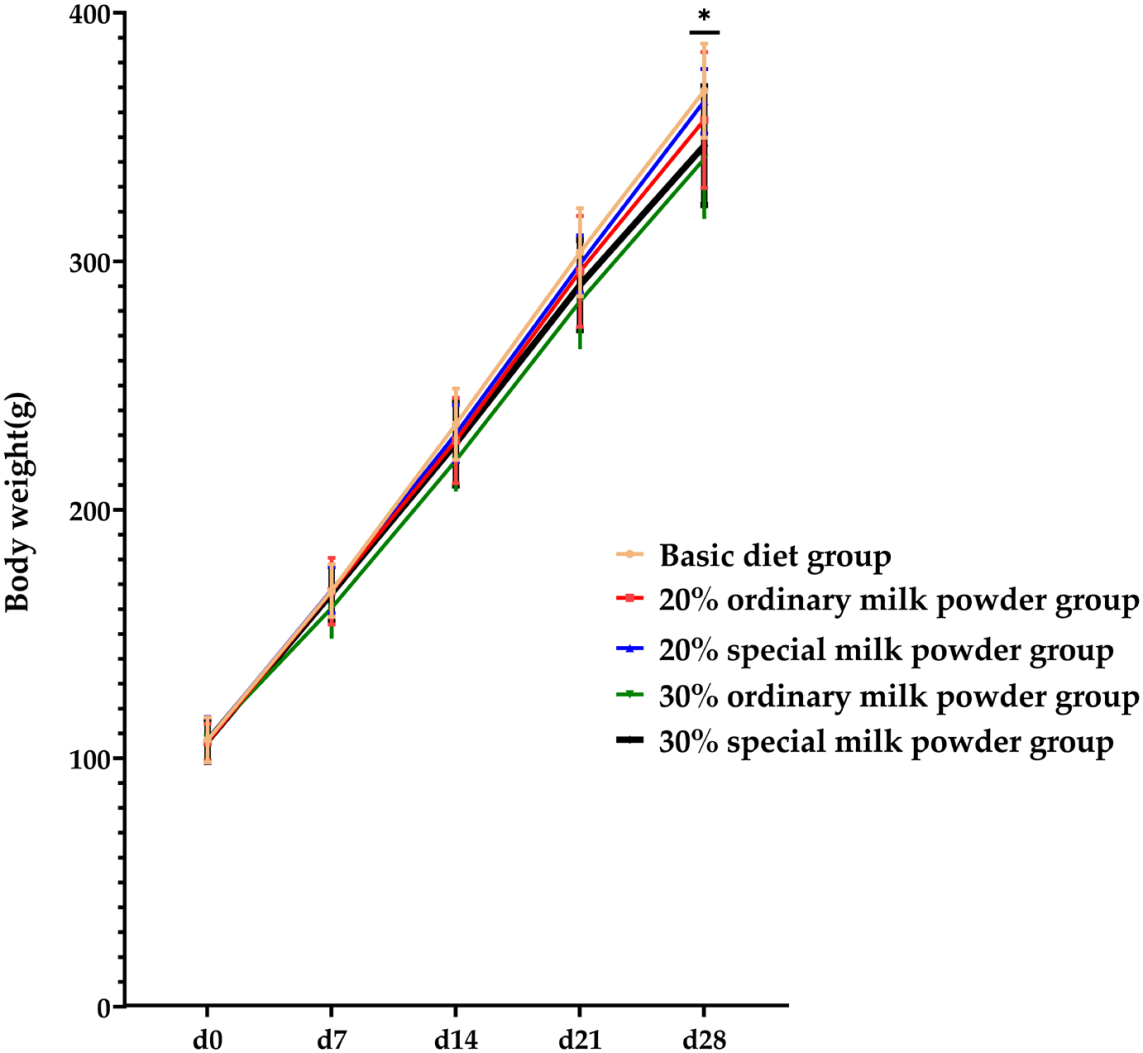
The body weight of rats feeding with ordinary milk powder and special formula milk powder (*n* = 10). *P〈0.05,**P〈0.01,***P〈0.005.

**FIGURE 3:**
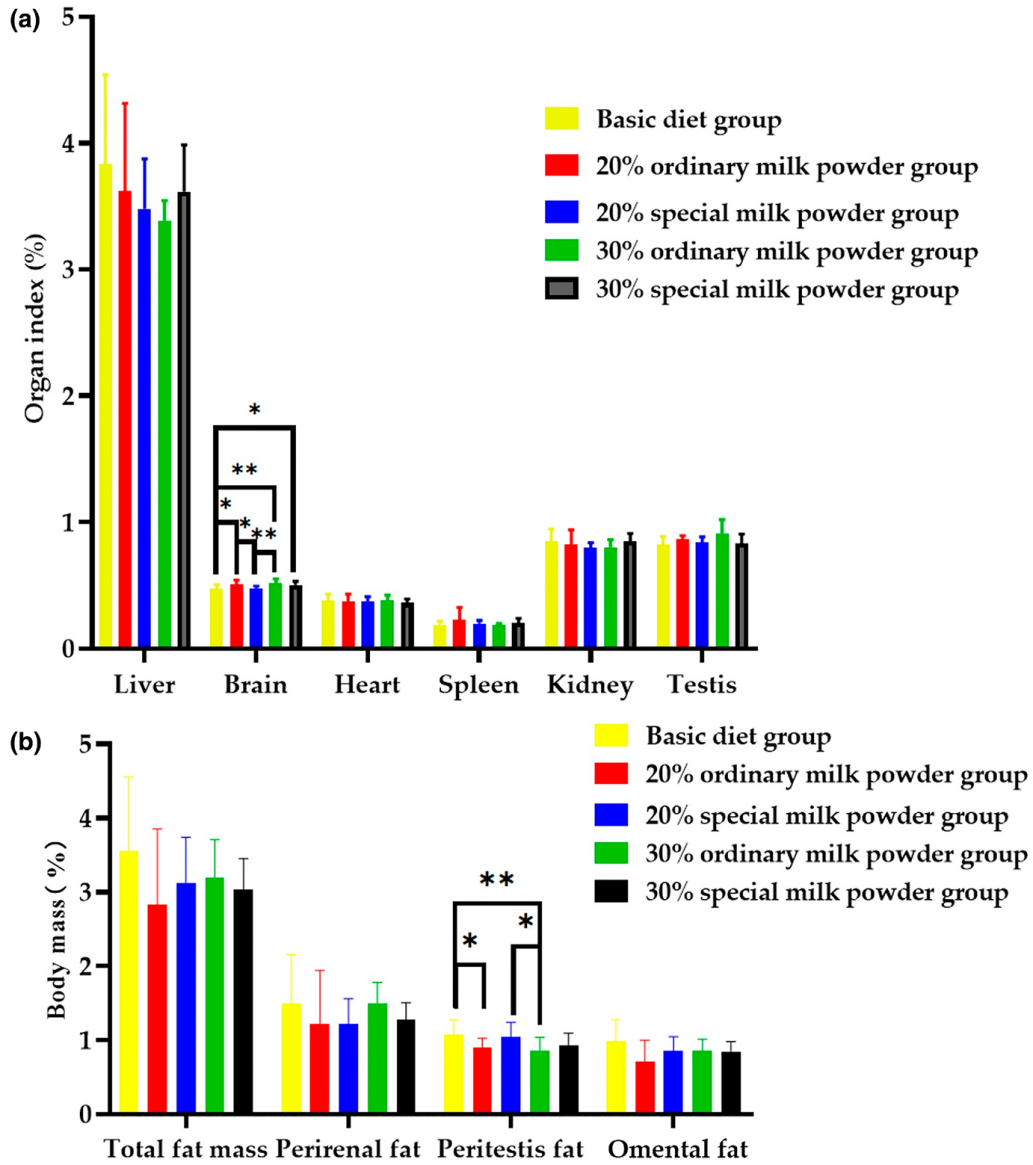
The organ coefficient and the body fat content of rats feeding with ordinary milk powder and special formula milk powder (*n* = 10). (a) Organ coefficient; (b) body fat content. *P〈0.05,**P〈0.01,***P〈0.005.

As shown in Figure 2a, after 28 days of group feeding, compared with the basic diet group, the body mass of rats in the 30% ordinary milk powder group reduced, and the difference was statistically significant (*p* = .008), and compared with the 20% special formula milk powder group, the body mass of rats in the 30% ordinary milk powder group was decreased (*p* = .023). Compared with the basic diet group, the body mass of rats in the 30% special formula milk powder group decreased, and the difference was statistically significant (*p* = .029). As shown in Figure 3a, brain weight/body weight of rats in the group supplemented with 20% ordinary milk powder increased compared with that in the basic diet group, with statistical significance (*p* = .019). Compared with the basic diet group, the brain weight/body weight of rats in the 30% ordinary milk powder group increased, and the difference was statistically significant (*p* = .004). Compared with the basic diet group, the brain weight/body weight of rats added 30% special formula milk powder increased, and the difference was statistically significant (*p* = .034). Compared with the 20% ordinary milk powder group, the brain weight/body weight of rats added 20% special formula milk powder decreased, and the difference was statistically significant (*p* = .035). Compared with the 30% ordinary formula milk powder group, the brain weight/body weight of rats added 20% special formula milk powder decreased, and the difference was statistically significant (*p* = .008). There was no significant difference between the other organ indexes. Compared with the basal diet group, the peritestis fat of rats supplemented with 20% ordinary milk powder decreased, and the difference was statistically significant (*p* = .035). Compared with the basal diet group, the peritestis fat of rats supplemented with 30% ordinary milk powder decreased, and the difference was statistically significant (*p* = .008). Compared with 30% ordinary milk powder group, 20% special formula milk powder group reduced peritestis fat, and the difference was statistically significant (*p* = .017).

### 3.2 The structure of ileum and colon of SD rats

**FIGURE 4:**
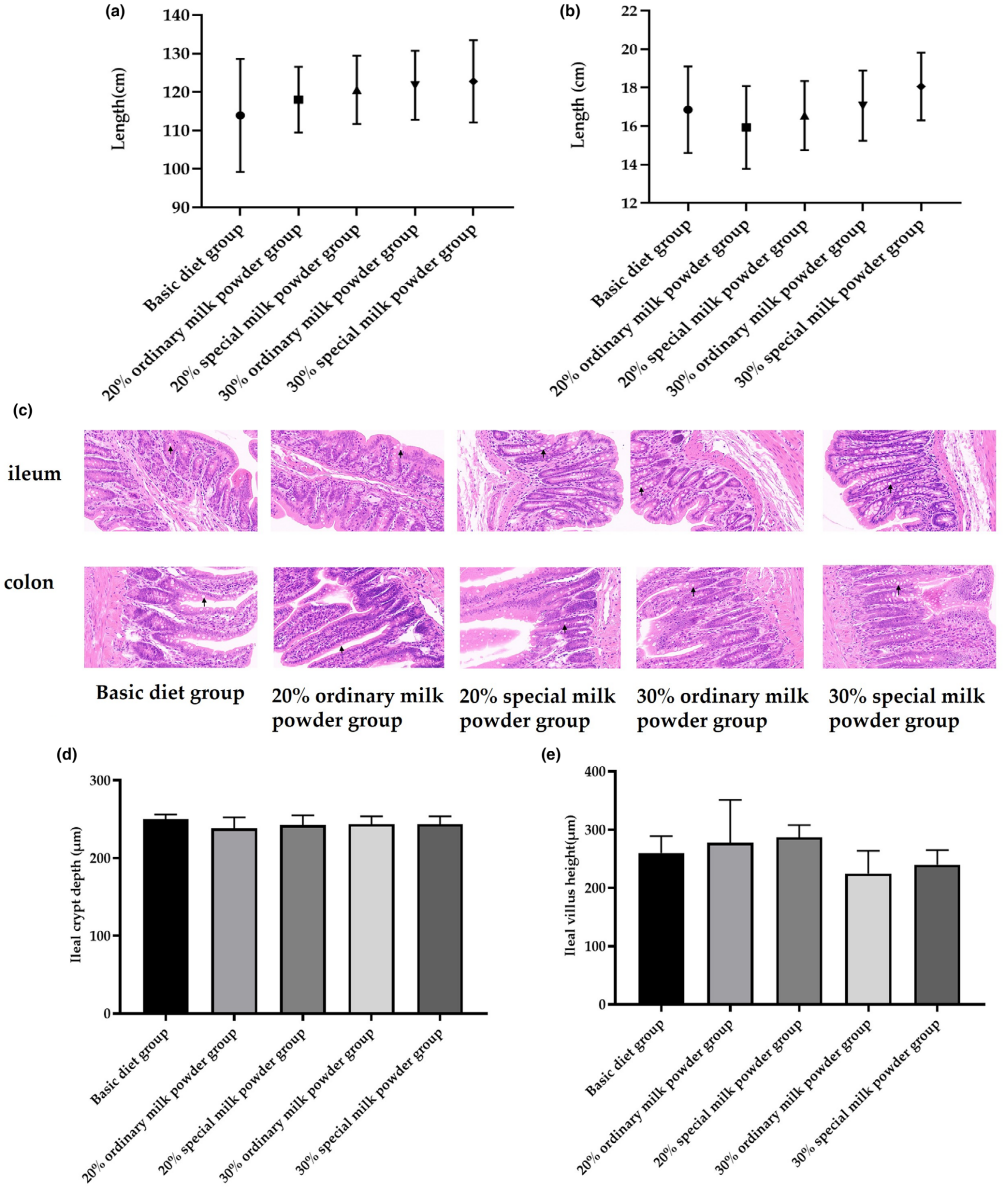
The structure of ileum and colon of rats feeding with ordinary milk powder and special formula milk powder (*n* = 3). (a) The length of colorectum; (b) small intestine; (c) the HE staining of colorectum and small intestine; (d) the ileal crypt depth of ileum; and (e) the ileal crypt height of ileum.

We also measured the length of small intestine and colorectum and the results showed no significant differences among these five groups (Figure 4a,b). As the results of hematoxylin and eosin (HE) staining showed in Figure 4c, the structure of ileum and colon was basically normal in each group, the intestinal villi were clear and complete in the visual field, the mucosal epithelial cells were closely arranged, and no necrosis was observed. The recesses were closely arranged with no obvious abscess or expansion. No edema was observed in submucosa. There was no obvious inflammatory cell infiltration in the tissue. We also measured the ileal crypt depth and the ileal crypt height of ileum and did not find statistical difference among groups.

### 3.3 Analysis of alpha diversity and beta diversity in the feces of SD rats

**FIGURE 5:**
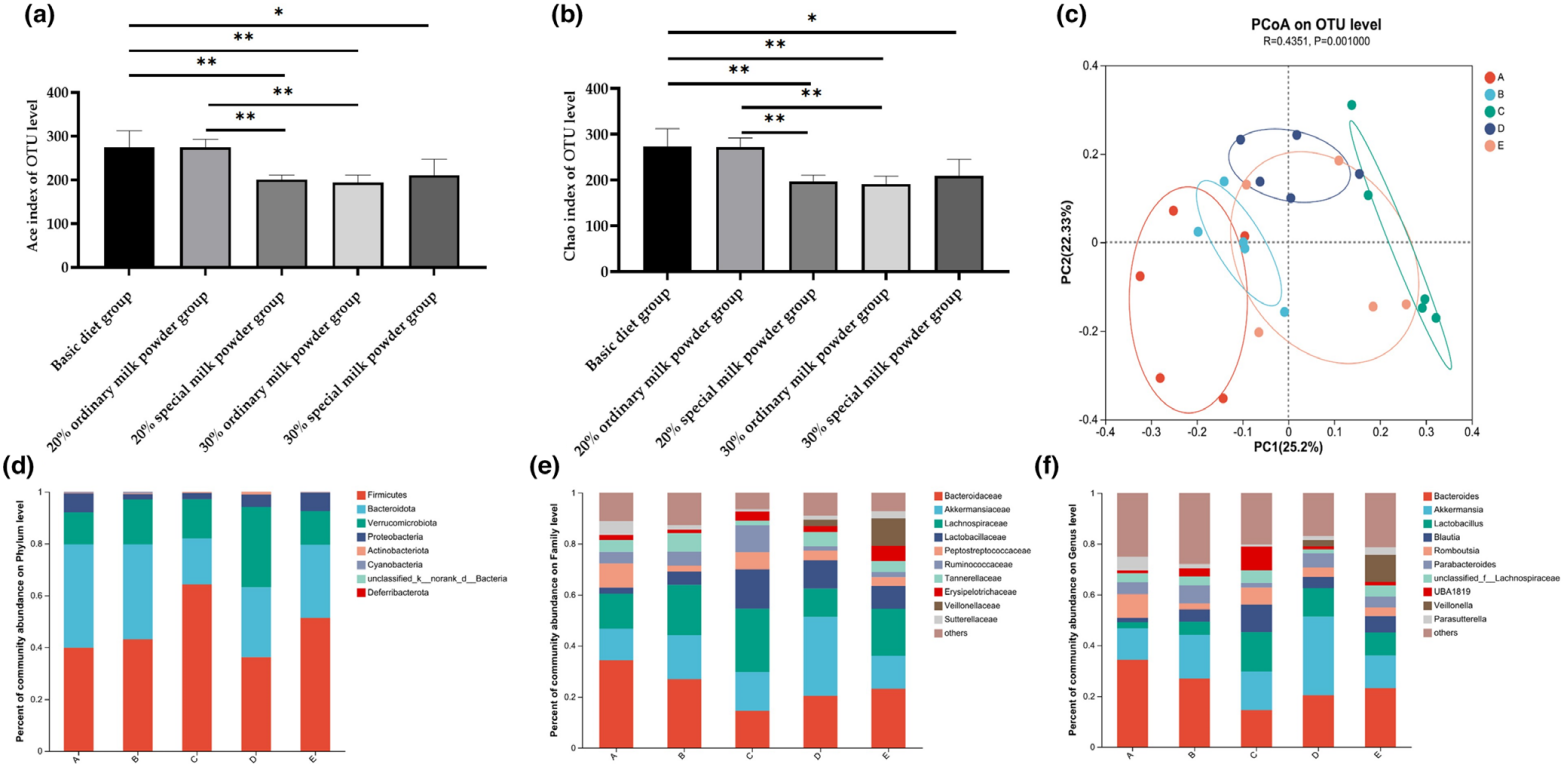
The intestinal flora of rats feeding with ordinary milk powder and special formula milk powder (*n* = 5). (a) The Ace index of OTU (Operational Taxonomic Units) level; (b) the Chao index of OTU level; (c) PCoA of OTU level; (d) the percent community abundance on Phylum level; e, the percent community abundance on Family level; the percent community abundance on Genus level. *P〈0.05,**P〈0.01,***P〈0.005.

As shown in Table S1, the sequencing coverage index of all samples was greater than 0.999, indicating that the probability of undetected microbial community in stool samples of SD rats was low. Therefore, the following results can reflect the real situation of the sequencing of this sample. Statistical analysis of Shannon index and Simpson index showed that there was no significant difference in intestinal flora diversity among SD rats among all groups (*p* > .05). As shown in Figure 5a,b, the Ace index and Chao index showed through statistical analysis that the intestinal flora abundance of SD rats in the basic diet group was significantly higher than those in the 20% special formula milk powder group (*p* = .003), 30% ordinary milk powder group (*p* = .003), and 30% special formula milk powder group (*p* = .039). The intestinal flora abundance of SD rats in the 20% ordinary milk powder group was significantly higher than those in the 20% special formula milk powder group (*p* = .008) and the 30% ordinary milk powder group (*p* = .008). Bray–Curtis' principal coordinate analysis (PCoA) was used to analyze the β‐diversity of fecal flora of SD rats in each group. As shown in Figure 5c, the intestinal flora of SD rats in each group had obvious independent clustering, and the intestinal communities had significant differences (*p* = .001). The first principal component (PC1) was 25.2%, and the second principal component (PC2) was 22.33%. At the Phylum level, Family level, and Genus level, the relative abundance of microbial populations in each group is shown in Figure 5d–f.

### 3.4 Species difference analysis in the feces of SD rats

**FIGURE 6:**
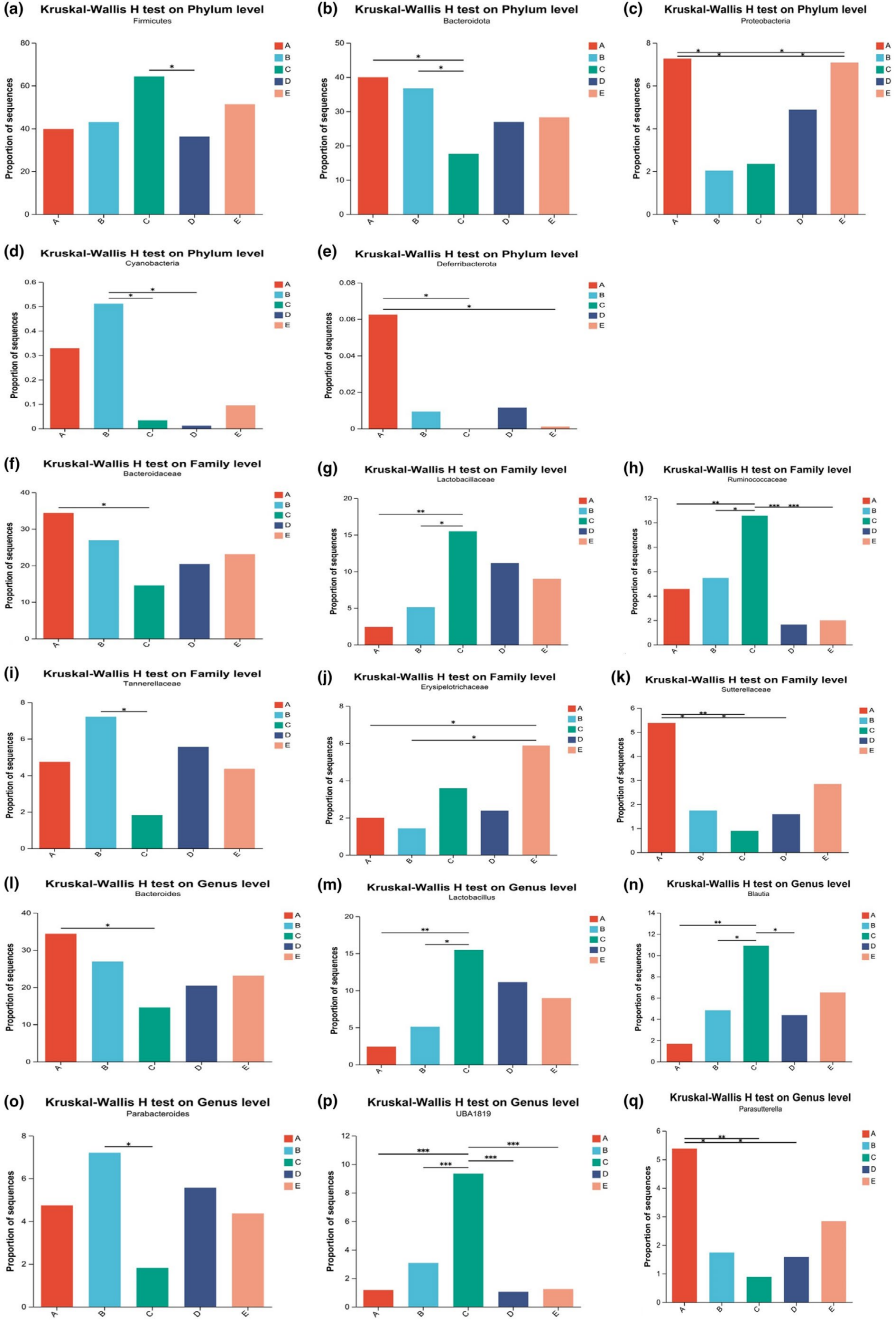
The difference analysis of intestinal flora of rats feeding with ordinary milk powder and special formula milk powder (*n* = 5). (a) *Firmicutes* differences on Phylum level among groups; (b) *Bacteroidota* differences on Phylum level among groups; (c) *Proteobacteria* differences on Phylum level among groups; (d) *Cyanobacteria* differences on Phylum level among groups; (e) *Firmicutes* differences on Phylum level among groups; (f) *Bacteroidaceae* differences on Family level among groups; (g), *Lactobacillaceae* differences on Family level among groups; (h) *Ruminococcaceae* differences on Family level among groups; (i) *Tannerellaceae* differences on Family level among groups; (j) *Erysipelotrichaceae* differences on Family level among groups; (k) *Sutterellaceae* differences on Family level among groups; (l) *Bacteroides* differences on Genus level among groups; (m) *Lactobacillus* differences on Genus level among groups; (n) *Blautia* differences on Genus level among groups; (o) *Parabacteroides* differences on Genus level among groups; (p) *ruminococcus* (*UBA1819*) differences on Genus level among groups; (q), *Parasutterella* differences on Genus level among groups. *P 〈0.05,**P〈0.01,***P〈0.005.

To explore the community composition of intestinal flora of SD rats in each group, according to the community abundance data in the samples, Kruskal–Wallis H test was used to test the significance of differences among the dominant phyla, dominant family, and dominant genus in the stool samples of SD rats in each group, and the relative abundance of microbial populations in each group was compared. At the Phylum level (Figure 6a–e), *Firmicutes* in the 20% special formula milk powder group was significantly higher than that in the 30% ordinary milk powder group, and *Bacteroidota* was significantly lower than those in the basic diet group and the 20% ordinary milk powder group. The *Cyanobacteria* was significantly higher than those in the group supplemented with 20% special formula milk powder and the group supplemented with 30% ordinary milk powder. The *Proteobacteria* in basic feed group and 30% special formula milk powder group were significantly higher than those in 20% ordinary milk powder and special formula milk powder group. *Deferribacterota* in the basic diet group was significantly higher than those in the 20% and 30% special formula groups. At the Family level (Figure 6f–k), *Bacteroidaceae* in the 20% special formula milk powder group was significantly lower than that in the basic feed group, and *Tannerellaceae* in the 20% ordinary milk powder group was significantly lower than that in the basic feed group. The *Lactobacillaceae* family was significantly higher than those in the basic feed group and the 20% ordinary milk powder group, and the *Ruminococcaceae* family was significantly higher than those in other groups. The content of *Sutterellaceae* in the basic feed group was significantly higher than those in the groups supplemented with 20% ordinary milk powder, special formula milk powder, and 30% ordinary milk powder. The *Erysipelotrichaceae* in 30% ordinary milk powder group was significantly higher than those in 20% ordinary milk powder group and basic feed group.

At the Genus level (Figure 6l–q), *Bacteroides* in the group supplemented with 20% special formula milk powder was significantly lower than that in the basic diet group, and *Parabacteroides* was significantly lower than that in the group supplemented with 20% ordinary milk powder. *Lactobacillus* was significantly higher than those in the basal feed group and 20% ordinary milk powder group, *Blautia* was significantly higher than those in the basal feed group and 20% and 30% ordinary milk powder groups, and *ruminococcus* (*UBA1819*) was significantly higher than those in other groups. The number of *Parasutterella* defined in the basic diet group was significantly higher than those in the group supplemented with 20% ordinary milk powder, special formula milk powder, and 30% ordinary milk powder.

### 3.5 Levels of short‐chain fatty acid in SD rats

**FIGURE 7:**
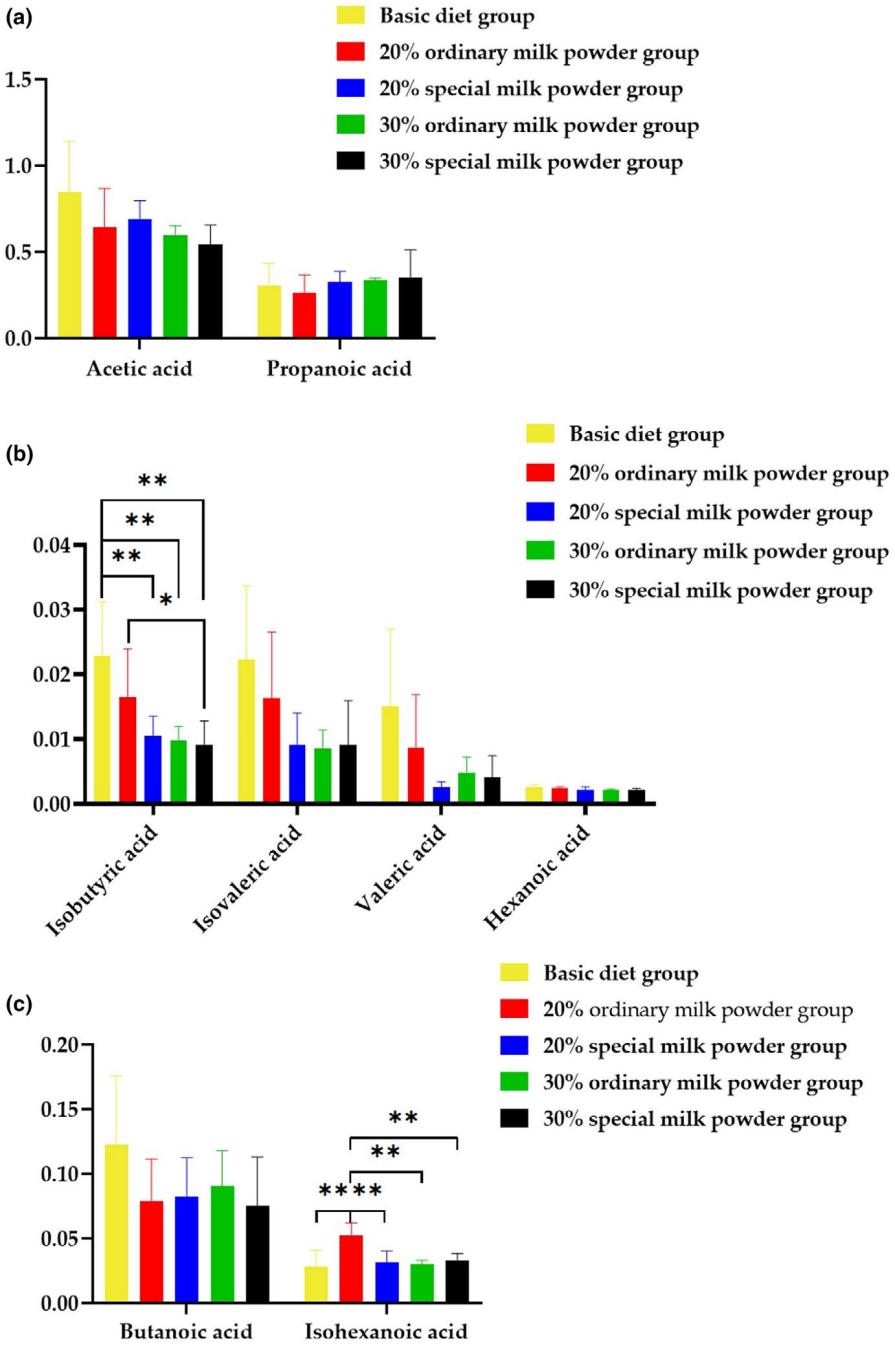
Levels of short‐chain fatty acid in SD rats feeding with ordinary milk powder and special formula milk powder (*n* = 5). (a) The acetic acid and propanoic acid levels among groups; (b) the isobutyric acid, isovaleric acid, valeric acid, and hexanoic acid levels among groups; (c) the butanoic acid and isohexanoic acid levels among groups. *P〈0.05,**P〈0.01,***P〈0.005.

Metabolites, such as acetic acid, propionic acid, isobutyric acid, butyric acid, isovaleric acid, valeric acid, isohexanoic acid, and hexanoic acid originating from gut microbiota, were quantified. Results showed that isobutyric acid and isohexanoic acid varied among these five groups.As shown in Figure 7b,c compared with basic diet group, the level of isobutyric acid was decreased in different doses of ordinary milk powder and special formula milk powder. While compared with 20% ordinary milk powder, the level of isobutyric acid was decreased in 30% special formula milk powder. The level of isohexanoic acid was significantly lower in control, 30% ordinary milk powder, and different doses of special formula milk powder than in the group supplemented with 20% ordinary milk powder (Figure 7c).

## 4 DISCUSSION

Adequate nutrition in early childhood is essential to ensure children's health, growth, and development (Brink et al., 2019; Moukarzel et al., 2018). Malnutrition is directly or indirectly responsible for 60% of infant and young child deaths worldwide each year, often due to improper feeding in the beginning of life (Li et al., 2019; Timby et al., 2014). Breast milk is acknowledged as the superior choice for nourishing babies, delivering readily absorbable components and essential nutrients in a well‐rounded manner that guarantees ideal growth and development in newborn babies (Lyons et al., 2020). All available evidence consistently indicates that breastfeeding provides clear advantages to newborns and infants, particularly through its influence on the composition of the gut's commensal microbiota (Xu et al., 2024). Breast milk is the poster child for infant formula, which aims to mimic the composition and function of breast milk by providing the ingredients of breast milk explored in the latest research to provide a nutritional solution for infants who cannot get enough breast milk. The focus of this study is to explore the effects of different doses and different formulations of infant milk powder on the growth and development of weaned rats (Figure 7).

The effects of different doses and different formulations on the growth and development of SD rats were studied by measuring the body weight of SD rats. As can be seen from Figure 2, there was no significant difference in the initial body mass of the rats in five groups before the experiment, but there was a difference between the groups from the second week and this difference remained until the end of the experiment. The body weight of all groups of animals increased with the extension of time, and the body weight of the basal diet group increased the fastest. Compared with the basic diet group, the body mass of rats in the 30% ordinary milk powder group and the 30% special formula milk powder group was lower, and the difference was statistically significant. During critical periods of early development, accelerated growth has been demonstrated to have a negative impact on glucose tolerance (Azad et al., 2018), and accelerated growth in human infancy may promote insulin resistance, obesity, and hypertension (Huxley et al., 2000; Monteiro & Victora, 2005; Soto et al., 2003). The results of this study show that the addition of 30% ordinary milk powder and special formula milk powder in the basic diet can accord with the trend of weight gain in normal rats fed exclusively breast milk, reduce the risk of obesity, and is beneficial to the long‐term health of rats.

Fortified infant formula is an effective breast milk substitute for feeding preterm infants because of better growth parameters (Gupta et al., 2020). In order to investigate the effects of different doses and different formulations of infant milk powder on organ development and body fat content of SD rats, the organ index and body fat content of newborn rats fed different formulations were measured. The results showed that there was no significant difference in organ index of heart, liver, spleen, lung, kidney, and testis among all groups. There was no significant difference in colorectal length and ileum length among all groups. These results indicated that different doses and different formula components of infant milk powder had no effect on the development of organ and intestinal length in SD rats. However, the brain/body weight of rats in the 20% ordinary milk powder group, the 30% ordinary milk powder group, and the 30% special formula group increased compared with the basic diet group, indicating that the supplementation of milk powder is beneficial to the growth and development of the brain of weaned rats, and can increase the brain weight. Milk powder has a higher nutritional value compared to the basic diet and therefore has a beneficial effect on the growth of brain in rats. In terms of body fat content, the fat content of the added milk powder group was lower than that of the ordinary feed group, indicating that the addition of milk powder reduced the risk of obesity in rats.

Mammalian intestinal flora is symbiotic with host and is closely associated with the immunity, digestion, and metabolism of host (Aron‐Wisnewsky et al., 2021; Sebastián Domingo & Sánchez Sánchez, 2018; Zhou et al., 2020). The composition of gut microorganisms is affected by host's genetic background, food, drugs, lifestyle, and other factors (Bibbò et al., 2016; Cuevas‐Sierra et al., 2019; Francino, 2015). Researchers can normally study the host gut microbiota through the diversity of the fecal microbiota. At present, the human gut microbiota has more than 1000 species (Devaux et al., 2020). The emulsification and absorption of lipids from food ingested by the human body, as well as metabolic processes, cannot be achieved without the production of short‐chain fatty acids (SCFAs) by the intestinal microbiota. It is worth noting that bioactive metabolites produced by the microbiota in the gut can also function as neurotransmitters. Neurodevelopment is associated with intestinal flora development over time (Ronan et al., 2021). Although nerve cell migration and nerve bundle growth occur during fetal development, gliosis, synapse building, and myelination continue throughout infancy and adolescence. Sustained nerve cell development throughout infancy will be influenced by a variety of factors, one of which is gut flora. Imbalance in the gut microbiome may result in alterations in its metabolites, which impact the communication within the gut–brain axis system, immune function of the brain, inflammation in the central nervous system (CNS), and the integrity and function of the blood–brain barrier (BBB) (Ortega et al., 2022).

In this experiment, the intestinal flora species were different, maybe due to the special formula that refers to milk powder with an appropriate amount of DHA, ARA, galactooligosaccharide, 1,3‐dioleic acid‐2‐palmitate glycerol triglyceride, lutein, nucleotide, lactoferrin, casein phosphopeptides, phospholipid, Ganglioside (GD3), and *Bifidobacterium* animalis subsp. lactis bb‐12 (BB‐12). At the Phylum level, *Firmicutes*, *Bacteroidota*, *Verrucomicrobiota*, and *Proteobacteria* were the dominant bacteria in the intestinal microbial community of SD rats in each group. The 20% special milk powder group had an increase in *Firmicutes* relative to the 30% ordinary milk powder group. It has been observed that *Firmicutes* in the gut are equipped with a plethora of genes involved in the fermentation of dietary fiber. Additionally, their interactions with the intestinal mucosa have been shown to play a crucial role in maintaining homeostasis (Sun et al., 2023). At the Genus level, the *Lactobacillus* in the 20% special formula milk powder group is significantly higher than those in the basic feed group and the 20% ordinary milk powder group, while the *Lactobacillus*, which are best understood for their applications as probiotics (Xiao et al., 2021), and our results showed that the 20% special formula milk powder can maintain intestinal health by increasing the abundance of *Lactobacillus*. The results of a population‐based trial showed that the high presence of fecal lactobacilli was significantly correlated with the *lactoferrin* levels in the stools of breastfed infants (Vega‐Bautista et al., 2019), indicating that *lactoferrin* is beneficial for contributing to the establishment of the gut microbiota. Animal experiments have shown that consumption of a casein‐based diet increases intestinal *Lactobacillus* levels in rats (Zhao et al., 2022), and the results of another study showed that mice fed a 1,3‐dioleic acid‐2‐palmitate glycerol triglyceride diet displayed higher mean relative abundance of *Lactobacillus* compared to mice fed a regular chow diet (Wang et al., 2020). Moreover, population‐based cohort studies have shown that the addition of oligogalactose to infant formula promotes the growth of *Lactobacillus* in the gut, resulting in fecal characteristics similar to those of full‐term infants fed human milk (Ben et al., 2004). Therefore, the addition of these special nutrients in the formula helps to maintain the diversity of the intestinal flora of infants. The *Blautia* group supplemented with 20% special formula milk powder was significantly higher than those supplemented with basic diet group and 20% and 30% ordinary milk powder groups. In recent years, *Blautia* has been proved to improve the content of SCFAs, regulate the structure of intestinal flora, and thus alleviate inflammation. Other strains of the genus *Blautia* have lipid‐lowering and cholesterol‐lowering properties and can be used as potential probiotics. It was reported that DHA‐enriched diet can increase the relative abundances of *Blautia* in high‐fat diet‐induced non‐alcoholic fatty liver disease (NAFLD) (Qian et al., 2022). A prior investigation demonstrated that the addition of probiotics containing *Bifidobacterium* animalis subsp. lactis bb‐12 (BB‐12) increased the abundance of beneficial bacterial strains in the intestinal flora of extremely premature babies both during and following supplementation. (Plummer et al., 2021) and animal experiments also support this idea (Xiao et al., 2003). Therefore, the addition of probiotics to special formulas may increase the abundance of beneficial intestinal bacteria in infants and children, thereby promoting intestinal health (Aziz, Naveed, et al., 2023). The number of *Parasutterella* defined in the basic diet group was significantly higher than those in the group supplemented with 20% ordinary milk powder, special formula milk powder, and 30% ordinary milk powder. In the last few years, it has been widely recognized that *Parasutterella* may be involved in the development and progression of irritable bowel syndrome (IBS), and that the high prevalence of chronic intestinal inflammation in patients with IBS has been linked to the said genus (Chen et al., 2018). Therefore, the addition of ordinary milk powder and special formula milk powder can improve the intestinal function of rats. *Ruminococcus* (*UBA1819*) *and Ruminococcaceae* in the group supplemented with 20% special formula were significantly higher than those in the other groups. The soluble dietary fiber polydextrose promoted the growth of beneficial microbes including *Ruminococcaceae* and *ruminococcus* (*UBA1819*) in obese mice (Hu et al., 2021). Uridine, one kind of nucleotide, has been reported to promote the growth of *Ruminococcus* (Liu et al., 2021). Therefore, the production of the above beneficial bacteria, the difference between the special formula milk powder group and the basic diet group, and the significant reduction of possible pathogenic bacteria all indicate that the special formula may promote the growth and development of weaned rats. Furthermore, the special formula also decreased some harmful bacteria such as *sutterellaceae* associated with depression (Chen et al., 2023) and *Parasutterella*, a bacterium possibly involved in the initiation and progression of IBS (Chen et al., 2018). Therefore, the nutrients including DHA, ARA, galactooligosaccharide, etc., added to special milk formulas may increase the beneficial bacteria in the intestinal flora of rats and may reduce the pathogenic bacteria.

Existing research suggests that SCFAs may be a crucial link between the gut microbiota and cognitive function (Chen et al., 2022). The findings of a cross‐sectional study conducted in Japan revealed a significant association between SCFAs and the incidence of dementia (Saji et al., 2020). The synthesis of SCFA is a product of the intricate interplay between the microbiota in the gut and the environment of the gut lumen, influenced by one's diet (Morrison & Preston, 2016). The main products of SCFAs are formate, acetate, propionate, and butyrate, and characterization of bacteria responsible for which can be identified by the metagenomic approaches. This study found that isobutyric acid and isohexanoic acid can increase intestinal permeability and may have a reverse effect on human health. In a notably sizable cohort of elderly individuals in Germany, researchers observed a correlation between isobutyric acid levels and the risk of developing Alzheimer's disease (AD) (Oluwagbemigun et al., 2023). Combined with the results of this study, it suggested that the special formula may decrease the levels of isobutyric acid and isohexanoic acid, protecting the intestinal mucosa.

## 5 CONCLUSION

This study found that different doses and different formula components of infant milk powder can affect the changes of food intake, body mass, brain weight index, and intestinal flora in SD rats. The addition of high dose (30%) of ordinary milk powder and special formula milk powder can reduce the food intake, decrease the body mass, and increase the brain weight of rats. The addition of low‐dose (20%) special formula infant milk powder can increase the beneficial bacteria in the intestinal flora of rats and may reduce the pathogenic bacteria.

## AUTHOR CONTRIBUTIONS

**Ruiqi Mu:** Data curation (equal); investigation (equal); project administration (equal); visualization (equal); writing – original draft (lead). **Yu Fu:** Data curation (equal); investigation (lead); software (equal); validation (equal); visualization (equal). **Jufang Li:** Formal analysis (equal); investigation (equal); methodology (equal). **Qinggang Xie:** Methodology (equal); resources (equal). **Weiwei Ma:** Conceptualization (lead); formal analysis (equal); methodology (lead); project administration (lead); resources (equal); supervision (lead); validation (equal).

## CONFLICT OF INTEREST STATEMENT

There are no conflicts to declare.

## Supporting information


Table S1

## Data Availability

The raw data supporting the conclusions of this article will be made available by the authors upon request.
