# Supplementary material for: Effects of different milk powders on the growth and intestinal flora in weaned rats: Comparison of special formula milk powder with ordinary milk powder
Source: Food Sci Nutr. 2024 Nov 12;12(12):10448–62. doi: 10.1002/fsn3.4387 (PMC11666978; doi:10.1002/fsn3.4387)
Supplement: Supplementary file 1 — Table S1 [file FSN3-12-10448-s001.docx]

Supplementary table 1. Analysis and comparison of intestinal microbiota diversity indexes

| Group | Ace index | Chao index | Shannon index | Simpson index | Coverage |
| --- | --- | --- | --- | --- | --- |
| A | 274.039±38.533 | 272.707±39.598 | 3.177±0.412 | 0.096±0.045 | 0.9993 |
| B | 274.594±19.019 | 272.570±19.684 | 3.375±0.290 | 0.076±0.033 | 0.9993 |
| C | 200.684±10.706 | 197.420±13.447 | 2.950±0.346 | 0.108±0.069 | 0.9995 |
| D | 194.564±17.089 | 191.223±17.335 | 2.751±0.217 | 0.141±0.048 | 0.9995 |
| E | 210.921±36.964 | 209.098±36.350 | 2.907±0.440 | 0.119±0.0713 | 0.9994 |

Supplementary table 1: Analysis and comparison of intestinal microbiota diversity indexes in the feces of SD rats in each group(n=5). A, basic diet group; B, 20% ordinary milk powder group; C, 30% ordinary milk powder group, D, 20% special milk powder group; E, 30% special milk powder group; Values are expressed as mean ± SD.
